# Shell-fish for Invalids

**Published:** 1893-06

**Authors:** 


### SHELL-FISH FOR INVALIDS.

Some of the different varieties of shell-fish are considered choice articles of food. They have a nutritive value a little less and somewhat similar to fish, but are not so easily digested. Oysters are the most easily digested of this class of food. Next come lobsters and crabs, and lastly mussels.

For invalids and dyspeptics, this class of food should be excluded from the diet, with the exception of oysters, and these for a person of weak digestion should be eaten raw, or merely warmed through, as cooking renders the oyster tough and more difficult of digestion.

The raw oyster agrees with almost every one, although a few find themselves obliged to discard the hard part, eating only the soft portion. The hard part is muscle, which binds the two half-shells together, and the soft is the liver.

